# Correction: The efficacy of pediatric elbow radiographic guidance in diagnosis of lateral humeral condyle fracture

**DOI:** 10.1371/journal.pone.0315028

**Published:** 2024-12-02

**Authors:** Satetha Vasaruchapong, Patarawan Woratanarat, Tanyaporn Patathong, Thira Woratanarat, Suphaneewan Jaovisidha, Chanika Angsanuntsukh

The fifth and sixth author names are spelled incorrectly. The correct names are: Suphaneewan Jaovisidha and Chanika Angsanuntsukh. The correct citation is: Vasaruchapong S, Woratanarat P, Patathong T, Woratanarat T, Jaovisidha S, Angsanuntsukh C (2024) The efficacy of pediatricelbow radiographic guidance in diagnosis of lateral humeral condyle fracture. PLoS ONE 19(3): e0300014. https://doi.org/10.1371/journal.pone.0300014.

There are errors in the author affiliations. The correct affiliations are as follows:

Satetha Vasaruchapong^1^, Patarawan Woratanarat^2^, Tanyaporn Patathong^2^, Thira Woratanarat^3^, Suphaneewan Jaovisidha^4^, Chanika Angsanuntsukh^2^

**1** Chakri Naruebodindra Medical Institute, Faculty of Medicine Ramathibodi Hospital, Mahidol University, Samut Prakan, Thailand, **2** Department of Orthopedics, Faculty of Medicine Ramathibodi Hospital, Mahidol University, Bangkok, Thailand, **3** Department of Preventive and Social Medicine, Faculty of Medicine, Chulalongkorn University, Bangkok, Thailand, **4** Department of Diagnostic and Therapeutic Radiology, Faculty of Medicine Ramathibodi Hospital, Mahidol University, Bangkok, Thailand.
