# Supplementary material for: The efficacy of pediatric elbow radiographic guidance in diagnosis of lateral humeral condyle fracture
Source: PLoS One. 2024 Mar 15;19(3):e0300014. doi: 10.1371/journal.pone.0300014 (PMC10942023; doi:10.1371/journal.pone.0300014)
Supplement: Attachment — Submitted filename: PONE-D-23-31280_reviewed.pdf [file pone.0300014.s001.pdf]

PLOS ONE

The efficacy of pediatric elbow radiographic guidance in diagnosis of lateral humeral condyle fracture

--Manuscript Draft--

**Manuscript Number:**	PONE-D-23-31280
**Article Type:**	Research Article
**Full Title:**	The efficacy of pediatric elbow radiographic guidance in diagnosis of lateral humeral condyle fracture
**Short Title:**	Lateral condyle fracture radiographic guidance
**Corresponding Author:**	Chanika Angsanuntsukh<br>Mahidol University Faculty of Medicine Ramathibodi Hospital<br>Bangkok, THAILAND
**Keywords:**	Lateral condyle fracture; humerus; diagnosis; radiograph; Guidance
**Abstract:**	Although lateral humeral condyle fracture is common, the incidence of missed diagnosis is very high. Delayed and missed diagnosis led to significant morbidities and loss of functions. We designed a pediatric elbow radiographic guidance aiming to improve the accuracy of diagnosis. This study was aimed to evaluate the efficacy of the radiographic guidance for the diagnosis of lateral condyle fracture. A cross- sectional study was conducted after defining the essential parameters as a guidance for assessing the pediatric elbow radiographs. We included medical students, emergency medicine, orthopedic, and radiology residents and fellows into this study. A questionnaire was used to evaluate the efficacy of the guidance. All participants underwent a pretest evaluation, followed by studying the guidance, and then finished a posttest evaluation. Baseline characteristics, diagnostic scores, and parameter evaluation scores were collected. The pretest and posttest scores were analyzed using paired t- test. Association between baseline characteristics and diagnostic scores were analyzed using multiple regression analysis. We included 177 participants. Average diagnostic score was significantly increased after using the guidance, from 12.2 + 1.9 to 13.0 + 1.7 ($p < 0.0001$). Medical students showed the most improvement, from 11.9 + 1.9 to 13.1 + 1.3 ($p <0.001$). All means of essential parameter evaluation scores were significantly improved in overall participants. The pediatric elbow radiographic guidance is useful for evaluation and diagnosis of lateral condyle fracture, especially for young physicians and trainees. Therefore, this should be recommended in routine medical education and general practice.
**Order of Authors:**	Satetha Vasaruchapong, MD
	Patarawan Woratanarat, MD,PhD
	Tanyaporn Patathong
	Thira Woratanarat, MD
	Supaneewan Jaovisidha
	Chanika Angsanuntsukh, MD
**Additional Information:**	
**Question**	**Response**
**Financial Disclosure**<br><br>Enter a financial disclosure statement that describes the sources of funding for the work included in this submission. Review the submission guidelines for detailed requirements. View published research articles from *PLOS ONE* for specific	The author(s) received no specific funding for this work.

Powered by Editorial Manager® and ProduXion Manager® from Aries Systems Corporation

examples.<br><br>This statement is required for submission and **will appear in the published article** if the submission is accepted. Please make sure it is accurate.<br><br>**Funded studies**<br>Enter a statement with the following details:<br>• Initials of the authors who received each award<br>• Grant numbers awarded to each author<br>• The full name of each funder<br>• URL of each funder website<br>• Did the sponsors or funders play any role in the study design, data collection and analysis, decision to publish, or preparation of the manuscript?<br><br>Did you receive funding for this work?	
**Competing Interests**<br><br>Use the instructions below to enter a competing interest statement for this submission. On behalf of all authors, disclose any competing interests that could be perceived to bias this work—acknowledging all financial support and any other relevant financial or non-financial competing interests.<br><br>This statement is required for submission and **will appear in the published article** if the submission is accepted. Please make sure it is accurate and that any funding sources listed in your Funding Information later in the submission form are also declared in your Financial Disclosure statement.<br><br>View published research articles from *PLOS ONE* for specific examples.	The authors have declared that no competing interests exist.

**NO authors have competing interests**<br><br>Enter: *The authors have declared that no competing interests exist.*<br><br>**Authors with competing interests**<br><br>Enter competing interest details beginning with this statement:<br><br>*I have read the journal's policy and the authors of this manuscript have the following competing interests: [insert competing interests here]*<br><br>* typeset	
**Ethics Statement**<br><br>Enter an ethics statement for this submission. This statement is required if the study involved:<br><br>• Human participants<br>• Human specimens or tissue<br>• Vertebrate animals or cephalopods<br>• Vertebrate embryos or tissues<br>• Field research<br><br>Write "N/A" if the submission does not require an ethics statement.<br><br>General guidance is provided below. Consult the submission guidelines for detailed instructions. **Make sure that all information entered here is included in the Methods section of the manuscript.**	This study was conducted after receiving approval from the Ethics Review Committee of Ramathibodi Hospital, affiliated with Mahidol University (ID 08-58-08). Each participant provided informed written consent before being included in the study.

Powered by Editorial Manager® and ProduXion Manager® from Aries Systems Corporation

**Format for specific study types**<br><br>**Human Subject Research (involving human participants and/or tissue)**<br>• Give the name of the institutional review board or ethics committee that approved the study<br>• Include the approval number and/or a statement indicating approval of this research<br>• Indicate the form of consent obtained (written/oral) or the reason that consent was not obtained (e.g. the data were analyzed anonymously)<br><br>**Animal Research (involving vertebrate animals, embryos or tissues)**<br>• Provide the name of the Institutional Animal Care and Use Committee (IACUC) or other relevant ethics board that reviewed the study protocol, and indicate whether they approved this research or granted a formal waiver of ethical approval<br>• Include an approval number if one was obtained<br>• If the study involved *non-human primates*, add *additional details* about animal welfare and steps taken to ameliorate suffering<br>• If anesthesia, euthanasia, or any kind of animal sacrifice is part of the study, include briefly which substances and/or methods were applied<br><br>**Field Research**<br><br>Include the following details if this study involves the collection of plant, animal, or other materials from a natural setting:<br>• Field permit number<br>• Name of the institution or relevant body that granted permission	
**Data Availability**<br><br>Authors are required to make all data underlying the findings described fully available, without restriction, and from the time of publication. PLOS allows rare exceptions to address legal and ethical concerns. See the PLOS Data Policy and FAQ for detailed information.	Yes - all data are fully available without restriction

A Data Availability Statement describing where the data can be found is required at submission. Your answers to this question constitute the Data Availability Statement and **will be published in the article**, if accepted.<br><br>**Important:** Stating 'data available on request from the author' is not sufficient. If your data are only available upon request, select 'No' for the first question and explain your exceptional situation in the text box.<br><br>Do the authors confirm that all data underlying the findings described in their manuscript are fully available without restriction?	
**Describe where the data may be found in full sentences. If you are copying our sample text, replace any instances of XXX with the appropriate details.**<br><br>• If the data are **held or will be held in a public repository**, include URLs, accession numbers or DOIs. If this information will only be available after acceptance, indicate this by ticking the box below. For example: *All XXX files are available from the XXX database (accession number(s) XXX, XXX.)*.<br>• If the data are all contained **within the manuscript and/or Supporting Information files**, enter the following: *All relevant data are within the manuscript and its Supporting Information files.*<br>• If neither of these applies but you are able to provide **details of access elsewhere**, with or without limitations, please do so. For example:<br><br>*Data cannot be shared publicly because of [XXX]. Data are available from the XXX Institutional Data Access / Ethics Committee (contact via XXX) for researchers who meet the criteria for access to confidential data.*<br><br>*The data underlying the results presented in the study are available from (include the name of the third party*	All relevant data are within the manuscript and its Supporting Information files

and contact information or URL).<br>• This text is appropriate if the data are owned by a third party and authors do not have permission to share the data.<br><br>* typeset	
Additional data availability information:	

Manuscript

Click here to access/download;Manuscript;PLOSOne_manuscript lateral

The efficacy of pediatric elbow radiographic guidance in diagnosis of lateral humeral condyle fracture

Satetha Vasaruchapong[1], Patarawan Woratanarat[2], Tanyaporn Patathong[2], Thira Woratanarat[3], Supaneewan Jaovisidha[4], Chanika Angsanuntsukh[2*]

[1]Chakri Naruebodindra Medical Institute, Faculty of Medicine Ramathibodi Hospital, Mahidol University

[2]Department of Orthopedics, Faculty of Medicine Ramathibodi Hospital, Mahidol University

[3]Department of Preventive and Social Medicine, Faculty of Medicine, Chulalongkorn University

[4]Department of Diagnostic and Therapeutic Radiology, Faculty of Medicine Ramathibodi Hospital, Mahidol University

Corresponding author

E-mail: chanika.ang@mahidol.edu

Abstract

Although lateral humeral condyle fracture is common, the incidence of missed diagnosis is very high. Delayed and missed diagnosis led to significant morbidities and loss of functions. We designed a pediatric elbow radiographic guidance aiming to improve the accuracy of diagnosis. This study was aimed to evaluate the efficacy of the radiographic guidance for the diagnosis of lateral condyle fracture. A cross-sectional study was conducted after defining the essential parameters as a guidance for assessing the pediatric elbow radiographs. We included medical students, emergency medicine, orthopedic, and radiology residents and fellows into this study. A questionnaire was used to evaluate the efficacy of the guidance. All participants underwent a pretest evaluation, followed by studying the guidance, and then finished a posttest evaluation. Baseline characteristics, diagnostic scores, and parameter evaluation scores were collected. The pretest and posttest scores were analyzed using paired t-test. Association between baseline characteristics and diagnostic scores were analyzed using multiple regression analysis. We included 177 participants. Average diagnostic score was significantly increased after using the guidance, from 12.2 ± 1.9 to 13.0 ± 1.7 ($p < 0.0001$). Medical students showed the most improvement, from 11.9 ± 1.9 to 13.1 ± 1.3 ($p < 0.001$). All means of essential parameter evaluation scores were significantly improved in overall participants. The pediatric elbow radiographic guidance is useful for evaluation and diagnosis of lateral condyle fracture, especially for young physicians and trainees. Therefore, this should be recommended in routine medical education and general practice.

Keywords: Lateral condyle fracture, humerus, diagnosis, radiograph, guidance

1. Introduction

Lateral condyle fracture is one of the most common elbow fractures in children[1]. The incidence is 1.6 per 10,000 children per year, which accounts for 12-20% of pediatric elbow fractures[2]. Even though lateral condyle fracture is very common, the diagnosis has been reported to be missed by clinicians. For instances, Shrader MW et al. reported that 60% of lateral condyle fracture were missed by general practitioners and emergency physicians[3].

Missed diagnosis of lateral condyle fracture can lead to non-union, malunion, limited range of motion, functional disturbance, varus/ valgus deformities and nerve palsy[2]. Some of these consequences result in permanent deformities and morbidity[4]. Therefore, accurate diagnosis and appropriate treatments are crucial for preventing the complications. To diagnose lateral condyle fracture, the physicians need to gather data from history taking, physical examination, and radiographic evaluation. Nevertheless, history taking and physical examination in young patients are challenging and sometime unreliable. Therefore, standard radiographs of the elbow are essential for the diagnosis[5]. Evaluation of pediatric elbow radiographs is difficult especially for trainees and general practitioners. Many essential radiographic parameters include posterior fat pad sign, anterior humeral line, radiocapitellar line, Baumann's angle and fracture line at lateral condyle on both anterior-posterior (AP) and lateral radiographs. Moreover, proper position of the elbow and quality of the radiographs are of import[6-10]

According to data from the medical council of Thailand, there are only 2,500 orthopedic surgeons in the country taking care of more than 69 million population[11, 12]. The proportion of

orthopedic surgeon per population is 1 per 27,600. Additionally, a large number of them are working only in big city areas. Therefore, the physicians who are early responsible for diagnosis of lateral condylar fracture are general practitioners due to limited numbers of orthopedic surgeon, but accurate diagnosis may be difficult and requires professional experiences. The authors initiated a radiographic guidance aiming to help the physicians to improve accuracy of diagnosis of lateral condyle fracture in children.

The purpose of this study was to evaluate the efficacy of the pediatric elbow radiographic guidance in diagnosis of lateral condyle fracture among inexperienced physicians and trainees, including medical students, emergency medicine residents, orthopedic residents and fellows and radiology residents and fellows.

2. Methods

Study design

A cross-sectional study was conducted after receiving approval from the Ethics Review Committee of Ramathibodi Hospital, affiliated with Mahidol University (ID 08-58-08). Each participant provided informed written consent before being included in the study. The study was conducted from September 2015 to September 2016.

2.1 Subject and Methods

We recruited medical students who have already finished orthopedic rotation, emergency medicine residents, orthopedic residents and fellows and radiology residents and fellows.

The literature reviews together with referencing the standard textbooks was done to identify the essential radiographical parameters for the diagnosis of elbow fractures in children. We then developed a pediatric elbow radiographic guidance explaining the definitions, providing the examples of normal and abnormal radiographs for each essential parameter. The steps for evaluating the radiographs were orderly exhibited. Starting with the first step is to assess the quality of the radiographs in both AP and lateral views, then look for the obvious fracture. If the fracture could not be clearly identified, the next step is to look for other five essential parameters to evaluate the radiographs including posterior fat pad sign, anterior humeral line, radiocapitellar line, fracture line in both AP and lateral views, and soft tissue swelling. These steps of five parameter evaluation were called "PARFS" (Posterior fat pad sign, Anterior humeral line, Radiocapitellar line, Fracture line, and Soft tissue swelling, respectively).

A questionnaire was created for pre-test and post-test evaluations in order to assess the efficacy of the guidance. The questionnaire consisted of 16 radiographs, including 5 normal cases, 2 supracondylar fractures, and 9 cases of lateral condyle fractures of various degrees. Details of radiographs such as patients' identification, serial numbers, data and time were blinded. All radiographs had been independently evaluated and confirmed for diagnosis by two experienced pediatric orthopedic surgeons. Each correct radiographic diagnosis was scored as 1 point, with a total possible score of 16 for the 16 radiographs.

After obtaining informed consent from the participants, we collected baseline characteristics, including age, gender, work experience, number of case experiences, orthopedic grade in medical school, and experience in orthopedic internship. Participants were then asked to

evaluate the questionnaire and record their evaluations on pre-test answer sheets. Subsequently, each participant was provided with the pediatric elbow radiographic guidance for 10 minutes of self-study. After finishing the study, they were asked to evaluate the questionnaire containing 16 radiographs in different orders from pre-test, and answered in post-test answer sheet. Baseline characteristics of participants, their radiographic evaluations, and diagnosis for each case from both pre-test and post-test were collected. We collected data in each department and included all participants who were willing to participate the study. There was no responders because all participants answer the questionnaire at the same time in department meeting room.

2.2 Statistical analysis

The sample size was calculated based on a pilot study of each group. The alpha error was 0.05 and power of the study was 0.8. Mean difference of pre-test and post-test diagnostic scores and standard deviations were 2.6 ± 2.39, 1.0 ± 1.92, 1.0 ± 1.09, and 0.79 ± 1.44 in medical students, emergency medicine residents, orthopedic residents and fellows and radiology residents and fellows, respectively. Sample size calculation were 9 for medical students, 31 for emergency medicine residents, 11 for orthopedic residents and fellows and 28 for radiology residents and fellows. Pre-test and post-test parameter evaluation scores and diagnostic scores were analyzed using paired t-test. The association between baseline characteristics and diagnostic scores improvement was tested using multiple regression analysis. The statistical analysis was done using STATA 13.0 (StataCorp, College Station, Texas, USA).

3. Results

One hundred and seventy-seven participants were enrolled in this study. There were 60 of medical students, 32 of emergency medicine residents, 57 of orthopedic residents and fellows, and 28 radiology residents and fellows. Baseline characteristics were significantly different in each group which represent the divergence of each group. Baseline characteristics were presented in **Table 1**.

Table 1. Baseline Characteristic

	n=177			
	**Medical student (n=60) (n)**	**Emergency medical student (n=32) (n)**	**Orthopedic resident and fellows (n=57) (n)**	**Radiology resiident and fellows (n=28) (n)**
Age				
< 25 years old	58	0	0	1
25-30 years old	1	30	34	24
>30 years old	1	2	23	3
Gender				
Female	37	21	6	24
Male	23	11	51	4
Work experience				
Studying	60	3	3	2
1 - 3 years	0	11	16	4
>3 years	0	18	38	22
Number of case experience				
< 5 cases	60	22	34	23
> 5 cases	0	10	23	5
Orthopedic grade in medical school year 5				
Grade A	4	2	8	1
Grade B, B+	25	11	30	6
Grade C, C+	4	5	14	3
Grade D,D+	1	0	0	0
Not remember	7	14	5	18

Orthopedic grade in medical school year 6				
Grade A	-	3	16	4
Grade B, B+	-	13	22	4
Grade C, C+	-	1	7	1
Grade D,D+	-	0	0	0
Not remember	-	15	12	19
Experience in orthopedic internship				
Yes	0	0	14	0
No	60	32	43	28

For the overall participants, mean of diagnostic scores was significantly improved from 12.4 ± 2.0 to 13.4 ± 1.4 after using the guidance (p = 0.003). Medical student was the most improved group, mean diagnostic score was improved from 11.9 ± 1.9 to 13.1 ± 1.3 (p <0.001), followed by emergency medicine (10.9 ± 1.8 to 11.8 ± 2.2, p < 0.001) and orthopedic residents and fellows (12.9 ± 1.6 to 13.7 ± 1.3, p = 0.002). For radiology residents and fellows, the mean of diagnostic scores was not significantly improved (12.5 ± 1.6 to 12.8 ± 1.5, p = 0.558) as shown in **Table 2**.

Table 2. Diagnostic score improvement

Group	Diagnostic score (mean(SD))		Mean difference (95%CI)	p-value
	Pre-test	Post-test		
Medical student	11.9 (1.9)	13.1 (1.3)	1.18 (0.66,1.70)	<0.001*
ER	10.9 (1.8)	11.8 (2.2)	0.94 (0.16, 1.71)	<0.001*
Orthopedics	12.9 (1.6)	13.7 (1.3)	0.75 (0.30, 1.21)	0.002*
Radiology	12.5 (1.6)	12.8 (1.5)	0.21 (-0.52, 0.96)	0.558
**Overall**	**12.4 (2.0)**	**13.4 (1.4)**	**1.0 (0.47, 1.52)**	**0.003***

*Significant (P<0.05), SD = standard deviation, CI = Confidence interval

Means of parameter evaluation scores were significantly improved for all four parameters in overall participants. The mean of posterior fat pad sign evaluation scores were increased from 11.2 ± 2.0 to 12.1 ± 1.7 (p < 0.001). Mean of anterior humeral line evaluation scores were improved from 11.0 ± 2.4 to 12.2 ± 2.0 (p < 0.001). For the mean evaluation scores of fracture line increased from 12.8 ± 1.7 to 13.5 ± 1.2 (p<0.001) in AP view, and 13.6 ± 1.8 to 14.2 ± 1.5 (p< 0.001) in lateral view. Finally, the mean of soft tissue swelling improved from 12.0 ± 2.1 to 13.11 ± 1.8 (p< 0.001) as shown in **Table 3**.

Table 3. Pre-test and post-test of radiographic parameters evaluation score

Radiographic evaluation (N = 177)	Pre-test score, mean (SD)	Post-test score, mean (SD)	Difference (95% CI)	p-value
Posterior fat pad sign	11.21 (2.09)	12.11 (1.71)	0.91 (0.57, 1.25)	<0.0001*
Medical student	10.38 (1.72)	12.32 (1.58)	1.93 (1.45, 2.42)	<0.0001*
ER	11.03 (2.03)	11.57 (1.96)	0.53 (0.47, 1.54)	0.2860
Orthopedic	12.03 (2.09)	12.14 (1.79)	0.11 (-0.49, 0.70)	0.7243
Radiology	11.46 (2.25)	12.21 (1.47)	0.75 (0.06, 1.56)	0.0676
Anterior humeral line	11.02 (2.37)	12.22 (2.04)	1.21 (0.85, 1.57)	<0.0001*
Medical student	10.43 (2.45)	12.15 (2.07)	1.72 (1.06, 2.38)	<0.0001*
ER	10.33 (2.20)	11.53 (2.10)	1.20 (0.13, 2.27)	0.0288*
Orthopedic	11.95 (1.95)	12.89 (1.84)	0.95 (0.44, 1.46)	0.0005*
Radiology	11.11 (2.62)	11.75 (2.01)	0.64 (0.30, 1.59)	0.1742
Radiocapitellar line	12.06 (3.01)	14.13 (1.86)	2.07 (1.66, 2.49)	<0.0001*
Medical student	12.03 (3.12)	14.02 (1.71)	2.17 (1.39, 2.95)	<0.0001*
ER	9.59 (2.54)	13.44 (2.10)	3.86 (2.84, 4.88)	<0.0001*
Orthopedic	13.25 (2.59)	14.07 (2.04)	0.82 (0.29, 1.36)	0.0031*
Radiology	12.25 (2.60)	14.82 (1.22)	2.57 (1.53, 3.61)	<0.0001*
Fracture line AP	12.78 (1.69)	13.49 (1.24)	0.71 (0.45, 0.97)	<0.0001*
Medical student	12.37 (1.99)	13.37 (1.35)	1.00 (0.47, 1.52)	0.0003*
ER	12.6 (1.71)	13.03 (0.96)	0.43 (-0.26, 1.13)	0.2144
Orthopedic	13.16 (1.40)	13.79 (1.25)	0.63 (0.20, 1.06)	0.0050*

Radiology	13.11 (1.29)	13.64 (13.22)	0.53 (0.11, 0.96)	0.0162*
Fracture line lateral view	13.66 (1.83)	14.20 (1.54)	0.54 (0.30, 0.78)	<0.0001*
Medical student	12.63 (2.04)	13.38 (1.68)	0.75 (0.32, 1.17)	0.0009*
ER	13.5 (1.50)	14.26 (0.98)	0.77 (0.16, 1.37)	0.0144*
Orthopedic	14.34 (1.43)	14.73 (1.36)	0.39 (0.05, 0.84)	0.0858
Radiology	14.64 (1.16)	14.78 (1.34)	0.14 (-0.37, 0.66)	0.5732
Soft tissue swelling	12.00 (2.14)	13.11 (1.81)	1.11 (0.78, 1.44)	<0.0001*
Medical student	11.08 (2.29)	12.51 (1.84)	1.42 (0.81, 2.04)	<0.0001*
ER	11.40 (2.04)	12.33 (1.75)	0.93 (0.01, 1.87)	0.0504
Orthopedic	12.70 (1.68)	13.66 (1.63)	0.96 (0.43, 1.49)	0.0005*
Radiology	13.18 (1.74)	14.11 (1.31)	0.93 (0.09, 1.77)	0.0310*

*Significant (P<0.05), SD = standard deviation, CI = Confidence interval

The multiple regression analysis of association between baseline characteristics and diagnostic scores showed no association as shown in **Table 4**. However, the increasing of age and work experience tend to improve the diagnostic scores with the coefficient 0.25 (-0.66, 1.15), and 0.13 (-0.47, 0.72), respectively.

Table 4. Multiple regression analysis for association between baseline characteristic and diagnostic score improvement

Variables	Coefficient	95%CI	Standard error	p-value
Age	0.25	-0.66, 1.15	0.45	0.591
Work experience (year)	0.13	-0.47, 0.72	0.30	0.668
Case experience (≥ 5 cases)	-0.03	-0.83, 0.77	0.41	0.936
5th year grade ≥ B	-0.13	-1.09, 0.82	0.48	0.780
6th year grade ≥ B	0.08	-0.86, 1.01	0.47	0.872
Orthopedic internship	0.32	-0.89, 1.53	0.61	0.715

*Significant (P<0.05), CI = Confidence interval

4. Discussion

This study demonstrated that pediatric elbow radiographic guidance significantly enhanced the ability to evaluate essential parameters and diagnose lateral condyle fractures.. Although there was no supportive data to indicate how many scores of improvement considered to be clinically significant, we can still pragmatically interpret that one-point improvement of the mean diagnostic score means one more patient accurately diagnosed for proper management

According to a previous study, the prevalence of missed diagnosis of lateral condyle fracture was as high as 60%.[3] In our study, the rate of missed diagnosis was 23% before using the guidance,. This may be from the use of the questionnaire with a specific answer format for essential parameters evaluation. The answer form might potentially guide the participants to evaluate all mandatory parameters and lead to the diagnosis, and the missed diagnosis would then be underestimated. Moreover, the final diagnosis was in binary form (yes or no), there was a possibility that the participant made a correct diagnosis by chance. These could be considered as a limitation of this study reflecting that using a questionnaire did not resemble the real situation.

In spite of the influential effect of the questionnaire to guide the participants and lower the rate of missed diagnosis in pre-test evaluation, the guidance still showed significant improvement of the diagnostic scores with the rate of missed diagnosis after using the guidance at only 16%. Participants' diagnostic scores of fracture line (AP view and lateral view) in Jacob type I were improved after reading the guidance. Participants who miss diagnostics of fracture line AP view in pre-test was 42.18% and post-test miss diagnostics was 14.50% (p-value<0.001). While, participants

who miss diagnostics of fracture line lateral view in pre-test was 16% and post-test miss diagnostics was 4.7% (p-value=0.006).

Even with the guidance use, the fracture was still missed by medical students, residents, and fellows. This pointed out that more diagnostic accuracy of lateral condyle fracture may need professional experience rather than only nourishing the explicit knowledge in radiographic evaluation. In order to foster our young health professionals, real case-based teaching, coaching, mentoring and continuing practice should be emphasized in our medical education system.

The strength of our study was the inclusion of various degrees of fracture severity as well as comparative levels of orthopedic knowledge and practical experience among participant groups. The limitation of this study included the study was conducted in one university hospital, the results may not conform to other levels of hospital. We provided only AP and lateral radiographs which were standard views in routine practice. However, the oblique radiographs was very important in diagnosing lateral condyle fracture. Further study should be done including the oblique radiographs. If the oblique radiograph is very helpful in diagnosis of lateral condyle fracture for general practitioner, then it should be introduce into routine practice as a standard radiographic view. Moreover, the questionnaire was radiographic based, lack of clinical information, which may alter decision making. A multicentered study with clinical and radiographic information should be done in the future.

5. Conclusion

The pediatric elbow radiographic guidance, using PARFS steps, is beneficial for radiographic evaluation and diagnosis, especially for low experienced physicians and trainees. It should be recommended for use in routine medical education and general practice.

Supporting information

S1. File. Figures.
(PDF)

Author Contributions

Conceptualization: Satetha Vasaruchapong, Patarawan Woratanarat, and Chanika Angsanunsukh

Data curation: Satetha Vasaruchapong, Patarawan Woratanarat, and Chanika Angsanunsukh

Formal analysis: Patarawan Woratanarat, Tanyaporn Patathong, and Chanika Angsanunsukh

Funding acquisition: Satetha Vasaruchapong and Chanika Angsanunsukh

Investigation: Satetha Vasaruchapong and Chanika Angsanunsukh

Methodology: Satetha Vasaruchapong, Patarawan Woratanarat, and Chanika Angsanunsukh

Resources: Patarawan Woratanarat, Supaneewan Jaovisidha, and Chanika Angsanunsukh

Software: NA

Supervision: Patarawan Woratanarat, and Chanika Angsanunsukh

Validation: Patarawan Woratanarat, and Chanika Angsanunsukh

Writing – original draft: Satetha Vasaruchapong, Tanyaporn Patathong, and Chanika Angsanunsukh

Writing – review & editing: Tanyaporn Patathong, Patarawan Woratanarat, Thira Woratanarat, and Chanika Angsanunsukh

References

1. V. Andrey, S. Tercier, F. Vauclair, A. Bregou-Bourgeois, N. Lutz, P. Y. Zambelli. Lateral condyle fracture of the humerus in children treated with bioabsorbable materials. Sci World J. 2013;2013:869418.

2. N. Tejwani, D. Phillips, R. Y. Goldstein. Management of lateral humeral condylar fracture in children. The Journal of the American Academy of Orthopaedic Surgeons. 2011;19(6):350-8.

3. M. W. Shrader, M. D. Campbell, D. J. Jacofsky. Accuracy of emergency room physicians' interpretation of elbow fractures in children. Orthopedics. 2008;31(12).

4. S. V. Skak, S. D. Olsen, A. Smaabrekke. Deformity after fracture of the lateral humeral condyle in children. Journal of pediatric orthopedics Part B. 2001;10(2):142-52.

5. S. F. DeFroda, H. Hansen, J. A. Gil, A. H. Hawari, A. I. Cruz, Jr. Radiographic Evaluation of Common Pediatric Elbow Injuries. Orthopedic reviews. 2017;9(1):7030.

6. Sidney M Jacoby, Martin J Herman, William B Morrison, A Lee Osterman, editors. Pediatric elbow trauma: an orthopaedic perspective on the importance of radiographic interpretation. Seminars in musculoskeletal radiology; 2007: Copyright© 2007 by Thieme Medical Publishers, Inc.,.

7. R. S. Iyer, M. M. Thapa, P. C. Khanna, F. S. Chew. Pediatric bone imaging: imaging elbow trauma in children--a review of acute and chronic injuries. AJR American journal of roentgenology. 2012;198(5):1053-68.

8. Sharaf Ibrahim. Tachdjian's Pediatric Orthopaedics: from the Texas Scottish Rite Hospital for Children. Malays Orthop J. 2015;9(1):53-.

9. A. Knutsen, T. Avoian, S. L. Borkowski, E. Ebramzadeh, L. E. Zionts, S. N. Sangiorgio. Accuracy of radiographs in assessment of displacement in lateral humeral condyle fractures. Journal of children's orthopaedics. 2014;8(1):83-9.

10. S.L. Weinstein, J.M. Flynn. Lovell and Winter's Pediatric Orthopaedics. 7 th ed: Wolters Kluwer Health; 2014.

11. TheMedicalCouncilofThailand. Information of doctors who received a certificate from the Medical Council of Thailand 2003 [Available from: https://www.tmc.or.th/statistics.php.

12. Demographic statistics Population and Housing of Thai people [Internet]. 2022. Available from: http://statbbi.nso.go.th/staticreport/page/sector/th/01.aspx.

Supplementary file of figures

Click here to access/download
Supporting Information
S1 File. Supplementary figures.docx

Supplementary file of figures

Click here to access/download
Supporting Information
S1 File. Supplementary figures.pdf
