# Supplementary material for: The efficacy of pediatric elbow radiographic guidance in diagnosis of lateral humeral condyle fracture
Source: PLoS One. 2024 Mar 15;19(3):e0300014. doi: 10.1371/journal.pone.0300014 (PMC10942023; doi:10.1371/journal.pone.0300014)
Supplement: Attachment — Submitted filename: Response to reviewers.docx [file pone.0300014.s002.docx]

**Authors’ responses to reviewers**

**5. Review Comments to the Author**

Please use the space provided to explain your answers to the questions above. You may also include additional comments for the author, including concerns about dual publication, research ethics, or publication ethics. (Please upload your review as an attachment if it exceeds 20,000 characters)

**Response to reviewer #1:**

1. Introduction addressed the magnitude of the problem and the aim of the work properly

**Answer** Thank you so much for your comment.

2. Methods were descried in needed details, validated questionnaires and scientific guidance would have been an addition

**Answer** Thank you so much for your comment.

3. Results properly addressed with needed statistical data enlisted

**Answer** Thank you so much for your comment

4. Discussion configured the week points in a clear manner

**Answer** Thank you so much for your comment

5. References updates is recommended

**Answer** Thank you so much for your comment.

We have updated the recent references in the manuscript as the reviewer recommended. We added the reference number 2, 3, 4, 6, 17, 18, and 19 to the manuscript, as shown below.

2.	B. Wang, R. Gao, Z. Li, Z. Guo, Z. Ji, W. Li, et al. Early versus delayed treatment of lateral condylar fracture of the humerus with > 2 mm displacement in children: a retrospective study. J Orthop Surg Res. 2023;18(1):139.

3.	T. Martins, R. Marappa-Ganeshan. Pediatric Lateral Humeral Condyle Fractures. StatPearls. Treasure Island (FL) ineligible companies. Disclosure: Raghavendra Marappa-Ganeshan declares no relevant financial relationships with ineligible companies.: StatPearls Publishing Copyright © 2023, StatPearls Publishing LLC.; 2023.

4.	J. J. Masquijo, M. Sanchez Ortiz, A. Ponzone, L. Fernández Korosec, A. Arkader. Fractures of the lateral condyle of the humerus associated with elbow dislocation in children. A systematic review of the literature. Rev Esp Cir Ortop Traumatol. 2022;66(2):95-104.

6.	S. H. S. Tan, J. Dartnell, A. K. S. Lim, J. H. Hui. Paediatric lateral condyle fractures: a systematic review. Arch Orthop Trauma Surg. 2018;138(6):809-17.

17.	M. A. Poppelaars, D. Eygendaal, B. The, I. van Oost, C. J. A. van Bergen. Diagnosis and Treatment of Children with a Radiological Fat Pad Sign without Visible Elbow Fracture Vary Widely: An International Online Survey and Development of an Objective Definition. Children (Basel). 2022;9(7).

18.	B. Yao, J. Ko, B. Maheshwer, A. M. Dumaine, C. V. Nguyen, R. W. Liu. Assessment and Validation of Anterior Humeral Line for Diagnosing Supracondylar Humerus Fractures in the Pediatric Population. J Pediatr Orthop B. 2023;43(9):555-9.

19.	L. M. Fader, T. Laor, E. A. Eismann, R. Cornwall, K. J. Little. Eccentric Capitellar Ossification Limits the Utility of the Radiocapitellar Line in Young Children. Journal of pediatric orthopedics. 2016;36(2):161-6.

**Response to reviewer #2:**

The authors have submitted a manuscript in which they described an additional diagnostic approach in identifying lateral humeral condylar fractures.

**Strengths**

- The authors chose an ongoing medical concern that is related to the scarcity of orthopaedic specialists in Thailand that reflects upon the society in missing one of most common elbow fractures in a vast population of children in a developing country.
- Abstract and the introduction were descriptive, proper info regarding the rational, reason and the background for the research were listed, methodology is clear and descriptive. The info listed appears to be sound, the language is clear. Tables and figures are clear and descriptive.
- No self-citations were found. Oldest citation listed is dating 2001.

**Weaknesses**

1. The study was conducted at the interval between 2015-2016, its almost 8 years since the study was conducted. Why did the author/s wait all this time to get their results published?

**Answer** Thank you so much for your comment.

We have submitted the manuscript to many journals and has revised it as the reviewers recommended in order to improve the manuscript all along. We also have included this restriction in the discussion as one of limitations of the study, line 210-212.

“This study was conducted in 2016 which was many years ago, so it may lag of some updated information. However, the pattern of lateral condyle fracture seems to be the same with unchanged classification during these years”

2. Line 54: contains a spelling mistake.

**Answer** Thank you for your comment.

We corrected the misspelling in line 54.

from

“Moreover, proper position of the elbow and quality of the radiographs are of import”

to

“Moreover, the proper position of the elbow and quality of the radiographs are important”

3. Table 1: contains numerical discrepancy between the total numbers listed for med. School students and Orthopaedic grade in medical school year 5 “marked in red”

**Answer** Thank you for your comment.

We corrected the number of medical students who cannot remember orthopedic grade in medical school year 5 in Table 1, from 7 to 26.

from

to

4. Discussion is self-focused, no comparison to relevant similar approaches. References are a rather few and can be enriched with the discussion.

**Answer** Thank you so much for your comments.

We revised the discussion, added more relevant literatures and updated the references as showed in line 185-201.

5. No visual abstract seen

**Answer** Thank you so much for your comments.

We added the visual abstract and submitted with the revised manuscript.
